# Characterization of the complete chloroplast genome of medicinal plant *Rheum officinale* (Polygonaceae)

**DOI:** 10.1080/23802359.2019.1623102

**Published:** 2019-07-10

**Authors:** Yimin Li, Huan Li, Xiaobin Hei, Yuanmin Li, Hui Li, Jing Gao, Yonggang Yan, Mengmeng Liu, Gang Zhang

**Affiliations:** a College of Pharmacy, Shaanxi Qinling Application Development and Engineering Center of Chinese Herbal Medicine, Shaanxi University of Chinese Medicine, Xi’an, China;; b Institute of Bioinformatics and Medical Engineering, School of Electrical and Information Engineering, Jiangsu University of Technology, Changzhou, China;; c College of Traditional Chinese Medicine, Hebei University, Baoding, China

**Keywords:** *Rheum officinale*, chloroplast genome, herbal medicine

## Abstract

The complete chloroplast (cp) genome of *Rheum officinale* Baill. was determined based on the Illumina Sequencing data. The cp genome is 161,563 bp in length. The overall G + C content of the cp genome was 37.31%. The *R. officinale* cp genome contains 129 genes, including 84 protein-coding genes, eight rRNA genes (four rRNA species), and 37 tRNA genes (20 tRNA species). A neighbour-joining phylogenetic tree clarified that the cp genome of *R. officinale* was closely related to that of *R. palmatum* in Polygonaceae.

*Rheum officinale* Baill., a valuable medicinal herb, is widespread in Shaanxi, Sichuan, Hubei, Guizhou, Yunnan, and Henan provinces of China. The root and rhizome of called Rhei Radix et Rhizoma is a very important traditional Chinese medicine, possessing many pharmacological actions, such as purgative and cathartic, anti-oxidative, and anti-inflammatory, etc. (Yang et al. 2012). Herein, we conducted a high-throughput sequencing analysis of the complete chloroplast (cp) genome of *R. officinale*. Furthermore, phylogenetic tree analysis was determined using MEGA X (Kumar et al. 2018) with the neighbour-joining algorithm.

*Rheum officinale* seedlings were collected from A’Ba country, Sichuan province (N 31°36′45.8″, E 101°54′36.6″). A voucher specimen (Ro170820LY) was deposited at Shaanxi University of Chinese Medicine Herbarium. Total genomic DNA of fresh leaves from a single individual plant was extracted with EASYspin plus Total DNA Isolation Kit (Aidlab, Beijing, China) following the manufacturer’s instructions and 2 μg genome DNA was sequenced using Illumina Hiseq 2500 Sequencing Platform (Illumina, San Diego, CA) at Majorbio Biotechnologies Inc. (Shanghai, China).

In total, 1 Gb of 150-bp raw paired reads were retrieved and then quality-trimmed using CLC Genomics Workbench v11.0 (CLC Bio, Aarhus, Denmark) with standard settings. The cp genome of *R. officinale* was reconstructed with MITObim v1.8 (Hahn et al. 2013), and the cp genome of *R. palmatum* (GenBank accession KR816224) was set as the reference (Fan et al. 2016). The cp genome was annotated in the program DOGMA with default parameters (Lohse et al. 2007).

The *R. officinale* cp genome is a double-stranded circular DNA of 161 563 bp in length and deposited in GenBank under the accession of MK674495. Its structure is consistent with most cp genomes in various plant species (Fan et al. 2016; Sun et al. 2016), consisting of a pair of inverted repeats (IRs) (30,614 bp, respectively), a large single-copy (LSC) (86,983 bp), and a small single-copy (SSC) (13,352 bp). The overall AT content of the cp genome is 62.69%, indicating the typical AT-rich of plant cp genomes (Qian et al. 2013). The *R. officinale* cp genome contains 129 genes, including 84 protein-coding genes (PCG), eight ribosomal RNA (rRNA) genes (four rRNA species), and 37 transfer RNA (tRNA) genes (20 tRNA species). Most of the gene species occur as a single copy while 15 gene species occur in double copies, including all rRNA species, five tRNA species, and six PCG species.

A neighbour-joining phylogenetic tree constructed with 55 proteins-coding genes extracted from the cp genomes of 14 taxa suggested that *R. officinale* and *R. palmatum* are phylogenetically related to each other (Figure 1). The cp genome of *R. officinale* and its phylogenetic tree analysis clarified here should be useful for the population genetics, molecular ecology, molecular authentication, and evolution studies of genus *Rheum*.

**Figure 1.**
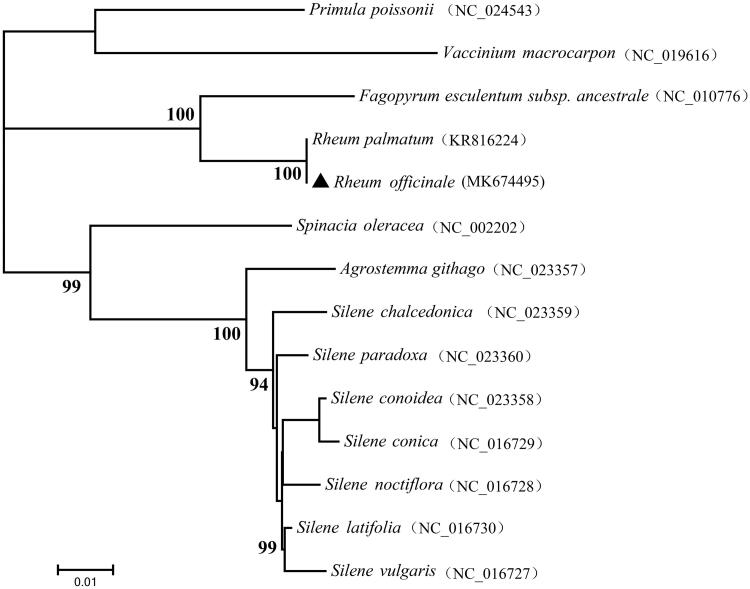
Phylogenetic relationships of *Rheum officinale* Baill. based on the neighbour-joining (NJ) analysis of 55 protein-coding genes in chloroplast genomes.
